# Fifty shades of partnerships: a governance typology for public private engagement in the nutrition sector

**DOI:** 10.1186/s12992-023-00912-1

**Published:** 2023-02-21

**Authors:** Dori Patay, Rob Ralston, Aliyah Palu, Alexandra Jones, Jacqui Webster, Kent Buse

**Affiliations:** 1 The George Institute for Global Health Australia, Sydney, Australia; 2 Global Heath Policy Unit, Social Policy, School of Social and Political Science, University of Edinburgh, Edinburgh, UK; 3 Healthier Societies Programme, George Institute for Global Health, Imperial College London, London, UK

**Keywords:** Multistakeholder engagement, Public-private partnerships, Food industry, Food policy, Food governance, Collaborative governance, Commercial determinants of health, Conflict of interest

## Abstract

**Background:**

Multistakeholder collaboration has emerged as a dominant approach for engaging and mobilising non-state actors; notably embedded in the paradigm of the UN Sustainable Development Goals. Yet, considerable ambiguity and contestation surrounds the appropriate terms of public private engagement (PPE) with industry actors.

**Main body:**

This paper seeks to conceptualise different forms of engagement with the food industry in tackling diet-related noncommunicable disease, within the context of power asymmetries across engaged stakeholders. It does so by introducing the Governance Typology for Public Private Engagement in the Nutrition Sector, a typology for government-led engagement with food industry actors across three domains: (i) the form of industry and civil society actor engagement (i.e., rules of exercising institutional power), based on the degree of participation in formal decision-making as well as participation at different stages in the policy cycle; (ii) the type of industry actors being engaged (i.e., pre-existing power attributes), based on function, size, and product portfolios for profit; and (iii) the substantive policy focus of engagement.

**Conclusions:**

The Governance Typology for Public Private Engagement in the Nutrition Sector seeks to inform national level nutrition policy makers on good engagement practice with food industry actors and complements existing risk assessment tools. This typology has the potential to inform decision-making on public sector engagement with other industries that profit from products detrimental to human and planetary health.

## Background

Diet-related noncommunicable diseases (NCDs) are responsible for the largest proportion of premature mortality globally [1]. Industries that profit from the sales of health harming commodities drive this NCD crisis [2]. The products and practices of the food industry exert a significant influence on population health and health equity [2]. While the effectiveness of mandatory regulation (e.g., taxation of sugar sweetened beverages) is well established [3, 4], non-binding ‘soft’ modes of regulation, particularly multi-stakeholder platforms and public-private partnerships, have emerged as widespread approaches at multiple levels of governance [5]. Within global governance, the United Nations Sustainable Development Goal 17 calls for the establishment of multistakeholder partnerships [6], with national-level governments launching multistakeholder platforms to tackle a range of climate and health challenges.

While multistakeholderism portrays and promotes collaboration between public and private actors as necessary and transformative in tackling NCDs, the legitimacy and effectiveness of this approach is contested [7, 8]. Although platforms engaging with industry to address issues such as food fortification have been implemented successfully, the wider literature points to stalled progress against the backdrop of increasing NCDs, competing interests and political contestation, particularly where proposed policies that address NCDs threaten core industry interests [2, 7, 8].

At least four factors have been put forward to explain why policy engagement to address diet-related NCDs with the food industry is often conflictual. First, nutrition as a field is multisectoral, including various government policy sectors from industry and trade to health and education, civil society, the food industry (and other associated industries, such as transport or public relations) and sometimes academic organisations [8, 9]. Moreover, it is multidisciplinary: it spreads over disciplines of food science and manufacturing, agriculture, public health, trade, education, economy, etc. [8, 9]. Such diversity inherently increases the likelihood of conflict as the actors often have contrasting worldviews, advance competing interests and promote different approaches to lowering the prevalence of diet-related NCDs [8, 9]. Second, reconciling conflicting interests of public and private actors can prove extremely challenging, given that the core business model of many food companies depends on maximizing profits from ultra-processed foods that are harmful to health [2, 8]. Third, the complexity of global and national nutrition governance makes reaching consensus among a variety of global actors and mechanisms challenging [8, 9]. Four, major power imbalances between private, public, and civil sector actors can render prioritisation of health interests difficult, resulting in a consensus that favours corporate interests and diminishes the health benefits of the collaboration [8, 10, 11]. Hence, policy engagement with the food industry can potentially serve to exacerbate and institutionalise existing power asymmetries [8, 10].

Despite the challenges posed by public private engagement to health equity, there are few indications that this paradigm of multistakeholderism will be challenged, with governments, international organisations, and industry actors remaining committed to collaboration. This reflects the prevailing idea that “the food industry is part of the solution” to solve the NCDs crisis [12]. This might be explained by the influence of large corporations on governments and multilateral organisations [13], the paradigm of ‘soft’ governance that has come to dominate transnational governance [14], and the notion that partnership and engagement between public and private actors can address market inefficiencies [15]. As a result, the value of collaborative approaches is often taken for granted by policy makers as they are perceived to be more effective, less costly and more straightforward to operationalize compared to mandatory regulation [5, 16].

Public private engagement (PPE) – the formal and informal governance processes through which state and non-state actors collectively work to design, promote, maintain and implement regulation – institutionalises the power stakeholders exercise over policy making. According to Moon’s [17] typology, policy actors may draw their power to influence governance from eight sources: economic power, through using material resources; institutional power, through established rules and processes in governance and decision-making; expert power, through being recognised for knowledge or skills; discursive power, by being able to shape the way others think; moral power, by being able to shape others’ moral principles; network power, through being able to harness the collective power of others; structural power, enabled the structures of society (e.g., governments); and physical power, through the use of force. Drawing on Moon’s typology [17], the institutional power granted by a PPE to private actors that may already hold considerable power from other sources (such as the economic power of transnational food manufacturers or the network power enjoyed by industry associations) can solidify these actors’ influence over public policies, which can be used to jeopardise public interests. However, the terms (i.e., conditions and forms) and governance arrangements of PPE will determine the level of institutional power formally granted to stakeholders and the extent to which that power can be exercised to undermine public policy goals in any given PPE [18].

Policy makers working within the prevailing paradigm of multistakeholderism are often confronted by institutional ambiguity about the appropriate terms of engagement with food industry actors. While the idea of partnership is synonymous with engaging the private sector, partnership itself may be used to describe varying types of engagement and does not necessarily reveal much about the involved parties’ respective responsibilities and decision-making authority (i.e., institutional power) [9]. Hawkes and Buse [9] propose to distinguish partnerships by the direction of funding (from private to public, from public to private, or joint funding), and whether decision-making authority is shared among participants or sectors. Others differentiate partnerships by the relationship between public and private actors, suggesting that their processes and outputs may be philanthropic, transactional, or transformational [19]. While this typology can help to understand the inter-organisational dynamics of partnerships, this approach may be less useful practically when proactively designing PPE. These typologies highlight the different forms PPE can take but could be complemented by more direct consideration of governance arrangements that can be embedded in policies from the start to enable government officials to make healthier public policy within multistakeholder approaches.

Tools have been developed to assist governments in considering whether to engage with particular food industry actors in advancing public health objectives. These include the draft World Health Organization approach on the prevention and management of conflicts of interest in the policy development and implementation of nutrition programmes at country level [20], complemented by a simplified triage tool [21]. While these resources suggest conducting a risk assessment for each individual actor, the public health community tends to discuss engagement with the food industry as if industry were a homogenous group of players, without differentiating the range of actors in this space [4]. Although the same groups of industry actors are often found across multistakeholder food platforms (e.g., trade associations, large transnational manufacturers), recognising the heterogeneity among industry actors is important in considering public health outcomes, as different types of actors (e.g. local fruit producers compared to industrial processors) are likely to have different products (and hence externalities), interests, resources, activities, sources and amount power, and thus might aim or able to influence population diet and food regulation differently [4]. This has led to a growing recognition among researchers that more analytical definitions of food industry actor types would help in conceptualising PPEs, which would be of use to policy makers and advocates [4].

In view of this, this paper seeks to conceptualise the forms that public sector engagement with the food industry can take, with particular focus on governance arrangements, taking into consideration the power resources at the disposal of and exercised by different stakeholders in these platforms. It does so by proposing the Governance Typology for Public Private Engagement in the Nutrition Sector, a typology for government-led PPEs established with industry actors to tackle diet-related NCDs. In this way, it seeks to inform policy makers and support them to consider a range of options for engaging on health policy matters with industry under the guise of “partnerships”.

### The governance typology for public private engagement in the nutrition sector

The consideration of three domains may help to better conceptualise PPE with the food industry to lower the prevalence of diet-related NCDs: (i) the form of food industry and civil society actor engagement, which shapes the ways institutional power can be exercised by stakeholders; (ii) the type of food industry actors engaged, reflecting pre-existing power attributes and interests; and (iii) the substantive policy focus of engagement, which offers additional considerations about the suitability of different stakeholders in PPEs. These domains are discussed in detail below and summarised in Table 1.

**Table 1:** The governance typology for public private engagement in the nutrition sector

**I. Form of food industry and civil society engagement (Rules on exercising institutional power)**	**(a) Degree of participation in formal decision-making (Extent of institutional power granted to stakeholders)**	Information provision
		Consultation
		Direct involvement
		Collaboration
		Empowerment
	**(b) Stage(s) of policy cycle (When and where institutional power can be exercised)**	Policy formulation
		Implementation
		Monitoring
		Evaluation
**II. Type of food industry actors engaged (Pre-existing power structures and interests)**	**(a) Product portfolios for profit (Interests)**	Profiting from sales of ultra-processed products
		Not profiting from sales of ultra-processed products
	**(b) Size (Economic power)**	Transnational companies
		Trade associations
		Large local companies
		Micro, small and medium-sized enterprises (MSMEs)
	**(c) Function (Other sources of power)**	Producers
		Manufacturers
		Distributors
		Retailers
		Hospitality
		Peak organisations
		Lobby and front organisations
**III. Substantive policy focus of engagement**		Public education and information
		Product reformulation
		Research
		Access to healthy foods
		Control of advertising and marketing
		Food product databases

A recent systematic review [7] assessed a range of PPEs with the food industry to improve population health. Based on this review, we applied the Governance Typology for Public Private Engagement in the Nutrition Sector (Governance Typology of Public Private Engagement, henceforth) to six PPEs that aim to address diet-related NCDs and were ranked as having high quality evaluations published. The domains of the Governance Typology for Public Private Engagement in relation to this select sample of PPEs is discussed below and summarised in Table 2.

**Table 2:** Analysis of PPEs based on the governance typology for public private engagement in the nutrition sector (MSMEs – micro, small, and medium sized enterprises)

PPE Project	Form of food industry and civil society engagement (Rules on exercising institutional power)				Type of food industry actors engaged (Pre-existing power structures and interests)			Substantive policy focus of engagement
	Degree of participation in formal decision-making (Extent of institutional power granted to stakeholders)		Stage(s) of policy cycle (When and where institutional power can be exercised)		Product portfolios for profit (Interests)	Size (Economic power)	Function (Other sources of power)	
	Food industry	Civil society	Food industry	Civil society				
**Food Standards Agency Strategy (2003–2010, United Kingdom)**	Direct involvement	Consultation	Policy formulation, Implementation, Monitoring	Policy formulation, Implementation	Actors that profit from sales of ultra-processed products	Transnational companies, local large, MSMEs	Manufacturers, retailers, caterers, peak organisations	Product reformulation, front-of-pack labelling, public awareness raising
**Public Health Responsibility Deal (Food) (2011–2015, United Kingdom)**	Collaboration	Collaboration	Policy formulation, implementation, monitoring	Policy formulation, monitoring	Actors that profit from sales of ultra-processed products	Transnational companies, local large, MSMEs	Manufacturers, retailers, caterers, peak organisations	Product reformulation, reduced portion size, new product development, responsible marketing and promotion, and disclosure of information.
**Less Salt, More Life (2009–2014, Argentina)**	Empowerment	Collaboration	Policy formulation, implementation	Policy formulation, monitoring	Actors that profit from sales of ultra-processed products	Transnationals, large local companies, MSMEs	Manufacturers	Product reformulation
**Food and Health Dialogue (2009–2015, Australia)**	Collaboration	Collaboration	Policy formulation, implementation, monitoring	Policy formulation, monitoring	Actors that profit from sales of ultra-processed products	Transnational companies, local large, MSMEs	Manufacturers, retailers, peak organisations	Product reformulation
**Healthy Food Partnership (2015 to present, Australia)**	Collaboration	Collaboration	Policy formulation, implementation, monitoring	Policy formulation, monitoring	Actors that profit from sales of ultra-processed products	Transnational companies, local large, MSMEs	Manufacturers, retailers, peak organisations	Product reformulation, public awareness raising.
**HOME GR/OWN (2013 to present, USA)**	Direct involvement	Direct involvement	Policy formulation, implementation	Policy formulation	Only actors that do not profit from sales of ultra-processed products	MSMEs	Producers	Access to fresh food

#### Form of food industry and civil society engagement

Existing typologies of PPE concentrate on organisational input and policy output and neglect the processes and procedures of decision-making and policy deliberation inherent in collaboration [19]. We outline two procedural dimensions to classify government-led PPEs based on the form of food industry and civil society engagement: (i) the degree of participation in formal decision-making, indicating the institutional power granted to stakeholders; and (ii) the stage(s) of the policy cycle they are engaged in, defining where and when the granted institutional power can be exercised.

The role of civil society actors is often to promote and safeguard public health interests and/or advocate for other policy outcomes [5, 22]. Considering the common power imbalance between food industry and civil society actors [8, 10], PPE may deepen this imbalance or potentially level the playing field by granting more institutional power to civil society and allowing them to exercise it more widely than industry actors in the policy making process. Therefore, in the design of PPE arrangements it is important to consider the rules on exercising institutional power for both industry and civil society actors.

##### Degree of participation in formal decision-making

Defining the industry and civil society actors’ degree of participation in formal decision-making helps understand the extent of institutional power the PPE will grant to these stakeholders. We drew from the International Association for Public Participation (IAP2) Spectrum of Public Participation [23] that was developed to assess civic participation in public policy making [23]. Applying the IAP2 framework to PPE is justified in our view as states and international organisations may ‘orchestrate’ governance arrangements with non-state actors, initiating and steering engagement with an industry actor, as the regulated party (or as co-regulator) [5, 21, 24]. The following differentiation of PPEs based on the stakeholders’ degree of participation in formal decision-making expands on Hawkes and Buse’s [9] framework.

According to the Governance Typology of Public Private Engagement, government agencies may engage with food industry and civil society actors in any of the following five ways: (i) *information provision* (provide information to enable compliance), (ii) *consultation* (obtain information and feedback on policy options, without the promise of those being consistently considered, and policy makers make all decisions); (iii) *direct involvement* (stakeholders are directly engaged in discussions with policy makers and their “concerns and aspirations are consistently understood and considered” [23] and systematically incorporated into the policy design, but policy makers make all decisions); (iv) *collaboration* (stakeholders and policy makers make decisions together); and (v) *empowerment* (government agencies allow stakeholders to make the decisions on aspects of PPE). At the risk of over-simplifying what may in practice constitute a continuum, in partnerships, the public sector typically offers collaboration or empowers private and civil society actors, while in public policy making, the public sector tends to inform, consult and directly involve other stakeholders.

We can use the above-identified categories to characterise PPEs (Table 2). For example, in the HOME GR/OWN initiative of the City of Milwaukee, United States, micro-, small- and medium-sized enterprises (MSMEs) specialised in urban farming and civil society organisations were *directly involved* in program development [25]. In contrast, in the case of the United Kingdom’s (UK) Public Health Responsibility Deal, the government and food industry actors *collaborated* in developing voluntary measures to improve population diet [26, 27]. The food industry actors had significant influence over the scope and content of the Deal due to being granted high levels of institutional power, while civil society actors reportedly had less influence as they received less institutional power by only being *directly involved* in the development of the voluntary measures [26, 27]. In the case of Argentina’s Less Salt, More Life PPE, food industry actors were *empowered* as they were granted autonomy to decide which food products to include in product reformulation [28]. As with the Public Health Responsibility Deal, civil society actors wielded less institutional power by being *directly involved* but not *empowered* in the way the food industry was [28]. These examples reveal that actors might be engaged in different ways within the same governance arrangement in a PPE due to underlying inter-organisational dynamics. These PPEs also highlight the importance of defining when, where and how non-governmental stakeholders are allowed to exercise institutional power.

##### Stage of the policy cycle

The terms and governance arrangements of PPE define what part of the policy making process stakeholders can influence through the exercise of their powers. The Governance Typology for Public Private Engagement suggests differentiating the form of food industry engagement according to the stages of the policy cycle where the engagement takes place [29]: (i) policy formulation (i.e., for the purposes of this paper, goal, rule and standard setting, or outlining specific measures for implementation); (ii) implementation; (iii)monitoring; and (iv) evaluation. Involvement in *policy formulation* entails food industry and civil society actors contributing to the development of the policy content. Taking part in *implementation* involves these actors having responsibility in executing aspects of the policy. Food industry actors’ participation in *monitoring* often includes the provision of quantitative or qualitative data on inputs, process and or outputs and outcomes. Civil society may be assigned a role of services delivery or take the opportunity to observe and *monitor* industry actors’ activities. Undertaking *evaluation* typically involves industry and/or civil society actors appraising the collaborative efforts in relation to the attainment of the PPE goals (as well as meeting their own interests). The *policy formulation* phase of PPEs is where the governance arrangements are developed, and this is the stage during which our proposed typology might be best applied. While the policy cycle approach may not reflect the messy realities of policy making, it offers an analytical lens that can help make sense of complex processes and in this case guide policy makers towards a well-considered public health enhancing PPE design.

Food industry interference in agenda setting, policy development and decision-making has been a major barrier in adopting mandatory food regulations in jurisdictions around the globe [2]. Voluntary, collaborative arrangements, such as PPEs, are often advocated for by industry as alternatives to mandatory measures in the agenda setting phase of the policy [7]. Thus, it is likely the case that by the time policy makers decide to engage with industry actors, the influence of these actors has already been exercised. We have not incorporated agenda setting into the typology, as its addition as a separate analytical stage would provide limited value given the aims of the typology – which are to aid in considering different PPE governance arrangements (i.e., after the idea for a PPE is already on the agenda). However, if the Governance Typology for Public Private Engagement was to be developed into a risk assessment tool, the incorporation of the agenda setting phase could raise its analytical value.

Australia’s Food and Health Dialogue provides a good example where food industry and civil society actors were involved in *formulating* voluntary measures on product reformulation [30, 31]. Industry actors were also responsible for *implementation* and participating in *monitoring* by providing self-reports, but they have had no role in *evaluating* the PPE to date [30, 31]. The UK’s Food Standards Agency sodium reduction strategy was government led, and food industry and civil society actors were consulted as part of *policy formulation* on technical issues related to the establishment of targets; *monitoring* combined both industry self-reporting and independent review [32]. Argentina’s Less Salt, More Life and the Milwaukee HOME GR/OWN initiative involved food industry actors in the *policy formulation* and *implementation* phases, but not in *monitoring* or *evaluation,* while the civil society had a role in policy *formulation* and *monitoring* [25, 28]. These examples demonstrate that engaging stakeholders at different stages of policy cycle may limit these actors’ ability to exert institutional power to jeopardise public health goals in PPEs (Table 2). Additionally, these examples reflect the need to consider stakeholders’ level and sources of power before enhancing their influence over PPEs.

#### Type of food industry actors engaged

Conceptualising the differences between the food industry actor types may help guide consideration about the form of engagement, by gaining a better understanding of how the governance arrangements of PPE enhance or limit pre-existing power structures. Earlier typologies of PPE do not typically incorporate a distinction between industry actor types [9, 19]. Following a pragmatic approach, the Governance Typology for Public Private Engagement suggests classifying food industry actors based on three factors: (i) product portfolios for profit, indicating their interests; (ii) size, indicating economic power; (iii) and function, potentially indicating other sources of power.

##### Product portfolios for profit

Considering the engaged stakeholders’ interests is important to predict whether the institutional power granted through the PPE might be used to undermine public health goals. The Governance Typology for Public Private Engagement recognises that food industry actors that profit from manufacturing and selling products that are not part of a healthy diet, such as ultra-processed foods and drinks, are likely to have different motivations and interests in participating in a PPE compared to those that profit primarily from the sales of healthy food items. Considering a food industry actor’s product portfolio is vital to any assessment of engagement, as it can forecast the likelihood of consensus on PPE goals and the potential for conflicts of interests.

For example, the HOME GR/OWN initiative engaged MSMEs that profit from selling fresh fruits and vegetables (Table 2) [25]. The aim of the PPE was to increase the consumption of these foods in the local community; therefore, the interests of these MSMEs were aligned with those of the PPE [25]. Thus, the government did not risk public health goals by granting institutional power to these otherwise less powerful food industry actors.

##### Size

The size of a company may indicate the extent of its economic power (e.g., through their assets and influence over markets and governments). The Governance Typology for Public Private Engagement categorises industry actors’ size based on number of employees and annual revenue, and whether the company pursues its primary activities in more than two countries. Industry actors can be categorised as micro-, small-, and medium-sized enterprises (MSMEs), large local enterprises, and transnational businesses [33]. Small-sized companies have fewer than 50 employees and a turnover of less than USD$10 million [33]. Medium-sized enterprises employ fewer than 250 workers with a turnover of up to USD$52 million, and large companies exceed these numbers [33]. Large enterprises with plants in a maximum of two countries are classed as large local enterprises, while those with plants in at least three countries are classified as transnational corporations.

Transnational corporations tend to be responsible for the majority of food manufacture and distribution in many countries, and governments commonly engage with them on food and nutrition initiatives [28, 30, 34–37]. Moreover, transnational corporations are often represented through trade associations in multistakeholder platforms and partnerships. For example, the above-mentioned PPEs in Argentina, Australia, and the UK all involved transnational corporations and trade associations [28, 30, 34–37]. These PPEs increased the influence of these already powerful food industry actors, potentially risking the achievement of public health goals.

Fewer PPEs engage with MSMEs (Table 2) [25, 28, 38]. The HOME GR/OWN initiative specifically focused on a MSMEs by connecting local food producers to community markets [25]. Although MSMEs have potentially less influence on the food environment than transnational companies [38], their collective impact on the food environment cannot be dismissed [22]. MSMEs are likely to provide more local employment than transnational companies and comprise most of the local food industry [39]. The HOME GR/OWN initiative enhanced MSMEs limited economic power by granting them institutional power which helped them advance these food industry actors’ interests (selling more fruit and vegetables) that align with the PPE aim (increasing fruit and vegetable consumption).

##### Function

Depending on their function, food industry actors may draw power from additional sources. For example, these may include networks (e.g., industry associations and peak organisations), expertise (e.g., manufacturers), or social structures (e.g., manufacturers and retailers) [17]. The Governance Typology for Public Private Engagement differentiates food industry actors based on the following categories or sectors: producers, manufacturers, distributors (including importers), retailers, hospitality, health and community (i.e., hospitals, nursing homes, childcare services), peak organisations, lobby and ‘front’ organisations. The retail sector in this typology covers supermarket chains and independent grocers. The hospitality industry includes restaurants and formal and informal street vendors.

Manufacturers are probably the most common food industry actors that governments engage with [7]; these actors tend to have structural and expert power based on their function, as their food formulation practices shape what people eat, and because they are often seen as the experts in food processing. For example, the Argentinian Less Salt, More Life initiative engaged with manufacturers [28] (Table 2). The UK Public Health Responsibility Deal, and Australia’s Food and Health Dialogue and Healthy Food Partnership involved mostly manufacturers and peak organisations (Table 2); these actors hold considerable expert, network and structural power. The power of these actors in these PPEs might explain some of the critique these initiatives have faced for failing to achieve intended public health goals [30, 34–37].

#### Substantive policy focus of engagement

Considering the amount of additional power PPE might grant to stakeholders and the ensuing risks to public health goals, the specific aims of different food related government initiatives might warrant the involvement of different types of private sector actors, which in turn might have implications for the most appropriate governance arrangements. Therefore, defining the focus of engagement in the PPE can be helpful to ensure that only appropriate actors are engaged. The Governance Typology for Public Private Engagement identifies the following categories based on existing food-related PPEs: (i) public education and information; (ii) product reformulation; (iii) research; (iv) access to healthy foods; (v) control of advertising, marketing and promotion; (vi) and establishing databases [10, 40, 41].

Product reformulation was the focus of Australia’s Food and Health Dialogue, Argentina’s Less Salt, More Life program [28] and The UK’s Food Standards Agency’s sodium reduction strategy (Table 2). The latter provides an example where reformulation was complemented with front-of-pack labelling and public awareness raising activities [32, 42]. The Public Health Responsibility Deal expanded this focus to responsible marketing and promotion and reducing portion size; although the parties could not reach agreement on any marketing measures [26, 27] (Table 2). These PPEs focused on policies that require a change in food industry practices, hence the inclusion of food manufacturers and retailers was likely warranted. On the other hand, the HOME GR/OWN initiative aimed to increase the accessibility and availability of fresh produce to communities; therefore, the government engaged with small primary producers [25] (Table 2). If, however, a PPE would primarily aim to increase consumer awareness on healthy diets, the involvement of most food industry actor types would not be justified, as potentially other actors, such as civil society organisations, could implement the initiative with lower likelihood for conflicts of interest. Therefore, involving food industry actors in PPEs that focus on changing the food environment is likely to be more beneficial than engaging them in knowledge-based activities to change dietary patterns [22, 30].

## Discussion

The Governance Typology for Public Private Engagement expands on existing typologies of PPE and draws on Moon’s [17] typology of power by showcasing the variety of governance arrangements multistakeholder initiatives can establish based on three domains: (i) form of food industry and civil society engagement, which defines the rules of exercising institutional power; (ii) type of food industry actors involved, indicating pre-existing power structures and interests; (iii) and the substantive policy focus of engagement, which offers additional considerations about the suitability of non-state participants. The Governance Typology for Public Private Engagement helps to consider the ways PPE may enhance the power of food industry actors and thus potentially undermining public health objectives. To the demonstrate its use, this paper applied the typology through the analysis of six PPEs.

The evidence on the effectiveness of voluntary, collaborative approaches to reduce diet-related NCDs is limited and contested [2, 7, 8, 22]. Furthermore, the factors relating to the institutional dynamic of regulation – the multisectoral and multidisciplinary nature of food policy, complexity of global and national food governance structures, and the conflicts of interest and power imbalance among policy actors – lead to considerable challenges to meeting public health goals through public private engagement [7, 8, 22]. The Governance Typology for Public Private Engagement helps clarify considerations about who is involved in any PPE, why they are involved and how, particularly in terms of pre-existing power assets and interests that should be considered when a PPE is designed. The typology is intended to help policy makers and advocates establish clear rules for relationships and avoid conflicts of interest that could otherwise diminish the potential beneficial public health impact of such arrangements. The typology also has the potential to inform PPE with other health harming industries that profit from products detrimental to planetary health, such as fossil fuels, agro-industrial chemicals, transport [30].

If policy makers are committed to food industry engagement, the Governance Typology for Public Private Engagement can help them to: (i) choose governance arrangements that limit industry actors’ participation in formal decision-making during certain stages of the policy cycle, and ensure that civil society actors have similar influence within the PPE through carefully considered rules governing the exercise of institutional power; (ii) narrow the type of food industry actors they engage by reflecting on pre-existing powers and interests; and (iii) avoid the engagement of food industry actors in focus areas where their participation is not justified or carries risk to public health goals.

Through a consideration of the form of food industry and civil society engagement, real and potential conflicts of interests between actors’ core activities and public health goals could be mitigated with less institutional power allocated to the food industry through lower degrees of participation. Although food industry actors tend to advocate for greater formal participation in decision-making in issues affecting their interests [5, 13], limiting their institutional power in PPEs can help to ensure that public health goals preside over private industry interests and thus make it more likely that the PPE can achieve its (public health) objectives.

In addition, PPE designs in which the form of industry involvement is limited to *consultation* or simply *providing information* tend to reduce the chance of undue industry influence on policy making [5, 7, 8, 19]. Limiting food industry engagement to the implementation stage might mitigate industry influence over the design of a PPE [7, 19]. Involving civil society actors in monitoring and evaluation might enhance the transparency and accountability of the PPE [2].

Regarding food industry types, as the size of a company is likely to correlate with its economic power, policy makers have a better chance to maintain a level playing field between stakeholders if transnational corporations are not involved [22]. Since MSMEs’ economic power is considerably more modest than transnational or large, local companies, involving them in a PPE would have potentially better chance to ensure more balanced power relations with potentially better health outcomes [4].

The consideration of the substantive policy focus of engagement may help policy makers ensure that the appropriate stakeholders are involved in the PPE. The involvement of food industry actors in initiatives focusing on public education and awareness raising creates an unnecessary risk for reaching the desired public health outcomes. Engaging food industry actors in activities that focus on changing the food environment might bring more benefits that those that concentrate on consumer knowledge-based activities [22, 30].

The Governance Typology for Public Private Engagement complements current risk assessment tools for industry engagement [20, 21] that guide policy makers in establishing nutrition policies at the national level. The typology highlights that the amount of risk a PPE with food industry actors carries depends on the combination of variables described above. For example, the optimal set up for a PPE that aims to *increase the availability and affordability of healthy, fresh foods* could be the following: engaging *small fruit and vegetable producers* through *direct involvement* in *policy formulation* an (as it has been demonstrated in the HOME GR/OWN initiative). This PPE design could be optimal, as the interests of these food industry actors align with the aim of the PPE, and these MSMEs’ limited economic power can be enhanced by reinforcing their institutional power to help achieve their common goal. However, policy makers should exercise caution in applying the same approach with *transnational manufacturers* whose interest is to profit from selling ultra-processed foods and thus not in line with the aim of most nutrition PPE. Instead, a PPE with *transnational manufacturers* could be beneficial if these actors are only *involved* in *implementation* (rather than policy formulation), as this would constrain these actors in using their power to influence the design and policy content of the PPE, affording them less opportunities to jeopardise public interests.

## Conclusion

Policy makers are urged to carefully consider whether PPE with the food industry is necessary and desirable, and if so, what form it ought to take. The Governance Typology for Public Private Engagement for Nutrition Sector provides a pragmatic way to conceptualise the governance arrangements and power structures of government-led multistakeholder initiatives involving the food industry. Its application may help policy makers to consider PPE designs that that do not necessarily entail a “partnership” and thus carry less risk to public health goals. Failure to systematically consider PPE design may invertedly undermine and/or delay efforts to tackle the NCD crisis.

## Data Availability

The datasets used and/or analysed during the current study are available from the corresponding author on reasonable request.

## Abbreviations

IAP2: International Association for Public Participation (IAP2)
MSMEs: Micro, small, and medium-sized enterprises
NCD: Noncommunicable disease
PPE: Public private engagement
UK: United Kingdom

## References

[1] Global Burden of Disease Collaborators (2020). Global burden of 369 diseases and injuries in 204 countries and territories, 1990–2019: a systematic analysis for the global burden of disease study 2019. Lancet.

[2] Buse K, Tanaka S, Hawkes S (2017). Healthy people and healthy profits? Elaborating a conceptual framework for governing the commercial determinants of non-communicable diseases and identifying options for reducing risk exposure. Glob Health.

[3] NCD Countdown 2030 Collaborators (2022). NCD Countdown 2030: efficient pathways and strategic investments to accelerate progress towards the Sustainable Development Goal target 3.4 in low-income and middle-income countries. Lancet.

[4] Lacy-Nichols J, de Lacy-Vawdon C, Moodie R, Maani N, Petticrew M, Galea S (2022). Defining the commercial determinants of health. The commercial determinants of health.

[5] Ralston R (2021). The informal governance of public-private partnerships in UK obesity policy: collaborating on calorie reduction or reducing effectiveness?. Soc Sci Med.

[6] UNGA. Resolution adopted by the general assembly on 25 September 2015 United Nations general assembly (UNGA); 2015. Contract No.: A/RES/70/1. https://www.un.org/en/development/desa/population/migration/generalassembly/docs/globalcompact/A_RES_70_1_E.pdf. Accessed 02 Jan 2023.

[7] Parker LA, Zaragoza GA, Hernandez-Aguado I (2019). Promoting population health with public-private partnerships: Where's the evidence?. BMC Public Health.

[8] Fanzo J, Shawar YR, Shyam T, Das S, Shiffman J. Challenges to establish effective public-private partnerships to address malnutrition in all its forms. Int J Health Policy Manag. 2021. 10.34172/ijhpm.2020.262. 10.34172/ijhpm.2020.262 PMC9309966 33619927

[9] Hawkes C, Buse K (2011). Public health sector and food industry interaction: it's time to clarify the term 'partnership' and be honest about underlying interests. Eur J Pub Health.

[10] Campos KDP, Cohen JE, Gastaldo D, Jadad AR (2019). Public-private partnership (PPP) development: toward building a PPP framework for healthy eating. Int J Health Plann Manag.

[11] Fuchs D, Lederer MML (2007). The power of business. Business Politics.

[12] Canfield M, Anderson MD, McMichael P. UN Food Systems Summit 2021: Dismantling democracy and resetting corporate control of food systems. Front Sustainable Food Syst. 2021;5. 10.3389/fsufs.2021.661552.

[13] Ralston R, Hil SE, da Silva GF, Collin J (2021). Towards preventing and managing conflict of interest in nutrition policy? An analysis of submissions to a consultation on a draft WHO tool. Int J Health Policy Manag.

[14] The United Nations Global Compact. The Ten Principles of the UN Global Compact, : The United Nations Global Compact; 2021 https://www.unglobalcompact.org/what-is-gc/mission/principles. Accessed on 21 Dec 2022.

[15] Sell SK, Williams OD (2020). Health under capitalism: a global political economy of structural pathogenesis. Rev Int Polit Econ.

[16] Johnston LM, Finegood DT (2015). Cross-sector partnerships and public health: challenges and opportunities for addressing obesity and noncommunicable diseases through engagement with the private sector. Annu Rev Public Health.

[17] Moon S. Power in global governance: an expanded typology from global health. Glob Health. 2019;15(S1). 10.1186/s12992-019-0515-5. 10.1186/s12992-019-0515-5 PMC6881906 31775779

[18] Buse K, Walt G (2000). Global public-private partnerships: Part I--A new development in health?. Bull World Health Organ.

[19] Kraak VI, Harrigan PB, Lawrence M, Harrison PJ, Jackson MA, Swinburn B (2012). Balancing the benefits and risks of public-private partnerships to address the global double burden of malnutrition. Public Health Nutr.

[20] WHO (2017). Draft approach for the prevention and management of conflicts of interest in the policy development and implementation of nutrition programmes at country level. Decision-making process and tool.

[21] PAHO (2021). Preventing and managing conflicts of interest in country-level nutrition programs: a roadmap for implementing the World Health Organization's draft approach in the Americas.

[22] Jones A (2019). Regulatory strategies to promote healthier diets.

[23] International Association for Public Participation. IAP2 Public Participation Spectrum: International Association for Public Participation; https://iap2.org.au/resources/spectrum/. Accessed on 21 Dec 2022.

[24] Bäckstrand K, Kuyper JW (2017). The democratic legitimacy of orchestration: the UNFCCC, non-state actors, and transnational climate governance. Environ Politics.

[25] Pettygrove M, Ghose R (2018). From “Rust Belt” to “Fresh Coast”: Remaking the City through Food Justice and Urban Agriculture. Ann Am Assoc Geographers.

[26] Panjwani C, Caraher M (2014). The public health responsibility Deal: brokering a deal for public health, but on whose terms?. Health Policy.

[27] Knai C, Petticrew M, Durand AM, Eastmure E, James L, Mehrotra A (2015). Has a public-private partnership resulted in action on healthier diets in England? An analysis of the public health responsibility Deal food pledges. Food Policy.

[28] Castronuovo L, Allemandi L, Tiscornia V, Champagne B, Campbell N, Schoj V (2017). Analysis of a voluntary initiative to reduce sodium in processed and ultra-processed food products in Argentina: the views of public and private sector representatives. Cad Saude Publica.

[29] Buse K, Mays N, Walt G (2012). Making health policy.

[30] Jones A, Magnusson R, Swinburn B, Webster J, Wood A, Sacks G (2016). Designing a healthy food partnership: lessons from the Australian food and health dialogue. BMC Public Health.

[31] Elliott T, Trevena H, Sacks G, Dunford E, Martin J, Webster J (2014). A systematic interim assessment of the Australian Government's food and health dialogue. Med J Aust.

[32] Wyness LA, Butriss JL, Stanner SA (2012). Reducing the population's sodium intake: the UK Food Standards Agency's salt reduction programme. Public Health Nutr.

[33] European Commission. Commission Recommendation of 6 May 2003 concerning the definition of micro, small and medium-sized enterprises. Document number C(2003) 1422. Brussels: European Commission; 2003. Contract No.: 32003H0361. https://eur-lex.europa.eu/legal-content/EN/TXT/?uri=CELEX:32003H0361. Accessed 02 Jan 2023.

[34] He FJ, Brinsden HC, MacGregor GA (2014). Salt reduction in the United Kingdom: a successful experiment in public health. J Hum Hypertens.

[35] Reeve B, Magnusson R (2015). Food reformulation and the (neo)-liberal state: new strategies for strengthening voluntary salt reduction programs in the UK and USA. Public Health.

[36] MacGregor GA, He FJ, Pombo-Rodrigues S (2015). Food and the responsibility deal: how the salt reduction strategy was derailed. BMJ..

[37] Coyle D, Shahid M, Dunford E, Ni Mhurchu C, McKee S, Santos M (2021). Estimating the potential impact of Australia's reformulation programme on households' sodium purchases. BMJ Nutr Prev Health.

[38] McKenzie B, Trieu K, Grimes CA, Reimers J, Webster J. Understanding barriers and enablers to state action on salt: analysis of stakeholder perceptions of the VicHealth salt reduction partnership. Nutrients. 2019;11(1). 10.3390/nu11010184. 10.3390/nu11010184 PMC6356996 30654526

[39] International Labour Organization (2019). Small matters – global evidence on contributions to employment by the self-employed, micro enterprises and SMEs.

[40] Hawkes C, Buse K. Public-private engagement for diet and health: addressing the governance gap. SCN News. 2011;39:6-10. ISSN : 1564-3743. https://www.cabdirect.org/cabdirect/abstract/20123084167. Accessed 02 Jan 2023.

[41] Larrick B, Kretser A, McKillop K. Update on “a Partnership for Public Health: USDA global branded food products database”. J Food Compos Anal. 2022:105. 10.1016/j.jfca.2021.104250.

[42] Magnusson R, Reeve B (2015). Food reformulation, responsive regulation, and "regulatory scaffolding": strengthening performance of salt reduction programs in Australia and the United Kingdom. Nutrients..

